# Retinal Thinning in Adults with Autism Spectrum Disorder

**DOI:** 10.1007/s10803-022-05882-8

**Published:** 2022-12-23

**Authors:** Evelyn B. N. Friedel, Ludger Tebartz van Elst, Mirjam Schäfer, Simon Maier, Kimon Runge, Sebastian Küchlin, Michael Reich, Wolf A. Lagrèze, Jürgen Kornmeier, Dieter Ebert, Dominique Endres, Katharina Domschke, Kathrin Nickel

**Affiliations:** 1 Department of Psychiatry and Psychotherapy, Medical Center – University of Freiburg, Faculty of Medicine, University of Freiburg, Freiburg, Germany; 2 Eye Center, Medical Center – University of Freiburg, Faculty of Medicine, University of Freiburg, Freiburg, Germany; 3 Faculty of Biology, University of Freiburg, Freiburg, Germany; 4 Institute for Frontier Areas of Psychology and Mental Health, Freiburg, Germany; 5 Center for Basics in Neuromodulation, Faculty of Medicine, University of Freiburg, Freiburg, Germany

**Keywords:** Autism spectrum disorder, ASD, Optical coherence tomography, OCT, Retina

## Abstract

Since the retina shares its embryological origin with the central nervous system, optical coherence tomography (OCT), an imaging technique frequently employed in ophthalmology to analyze the macula and intraretinal layer thicknesses and volumes, has recently become increasingly important in psychiatric research. We examined 34 autistic and 31 neurotypical adults (NT) using OCT. Autistic adults had reduced overall macular and outer nuclear layer (ONL) thickness and volume compared to NT. Both macular and ONL thickness showed significant inverse associations with the severity of autistic symptoms measured with the Social Responsiveness Scale 2 (SRS-2). Longitudinal studies across different age groups are required to clarify whether retinal changes may represent a possible trait marker.

**Supplementary Information:**

The online version contains supplementary material available at 10.1007/s10803-022-05882-8.

Autism spectrum disorders (ASD) are characterized by difficulties in social interaction and communication as well as stereotypical behaviors including hypo- and/or hypersensitivity to sensory stimuli (American Psychiatric Association, 2013; Tebartz van Elst, 2021). The underlying etiology is still unknown, with a strong genetic component, environmental and neurobiological factors as well as brain developmental alterations appearing to be involved (Lord et al., 2018; Pardo & Eberhart, 2007). Neuroimaging studies in ASD point towards an altered trajectory of brain maturation with an overgrowth of brain volume in infancy and early childhood followed by a normalization in later childhood and adolescence (Courchesne et al., 2011; Ecker et al., 2015; Prigge et al., 2021). Hox genes are important in the molecular control of early morphogenesis (Gokhale & Gokhale, 2007) and were suggested to be involved in the central nervous system (CNS) development with the *HOXA1* A218G polymorphism being associated with an enlarged cranial circumference in autism (Conciatori et al., 2004; Hashem et al., 2020). The retina and optic nerve share their neuroectodermal embryological origin with the brain, and are regarded as part of the CNS (Chua et al., 2021; Nguyen et al., 2017). Moreover, the thickness and volume of the intraretinal layers were found to be associated with total brain, gray and white matter volume (Chua et al., 2021; Mauschitz et al., 2022). Therefore, optical coherence tomography (OCT) as a non-invasive in vivo ophthalmologic routine examination method (Huang et al., 1991) is considered an innovative tool to investigate potential retinal alterations in autistic adults (Little, 2018; Nguyen et al., 2017; Silverstein et al., 2020) and can be regarded as a “window to the brain” (Schönfeldt-Lecuona et al., 2015).

OCT is a high-resolution imaging technique that relies on interference between the signal of an examined object and a local reference signal (Podoleanu, 2012) providing high-resolution cross-sectional images of the retina (Bagci et al., 2008). OCT has gained increasing importance in the examination of neurological disorders such as multiple sclerosis (Noval et al., 2011), Parkinson's and Alzheimer’s disease (Chan et al., 2019; Jindahra et al., 2010). In Alzheimer’s disease, for example, a thinner retinal nerve fiber layer (RNFL) was associated with an increased risk of dementia (Mutlu et al., 2018) and discussed as a possible correlate which might provide information on neurodegeneration of the brain (Mutlu et al., 2017). Therefore, OCT has also become a focus of psychiatric research, particularly in the investigation of patients with schizophrenia (Nguyen et al., 2017; Silverstein et al., 2020).

Studies investigating autistic adults applying OCT, however, are rare. Emberti Gialloreti et al. (2014) performed OCT examinations in 24 adults with ASD (11 with high functioning autism (HFA), 13 with Asperger Syndrome (AS)) compared to 24 neurotypical adults (NT). Autistic adults showed reduced thickness of the nasal peripapillary retinal nerve fiber layer (pRNFL) compared to NT. When dividing the ASD group into HFA and AS subsamples, the HFA group exhibited reduced global pRNFL thickness as well as nasal and inferior pRNFL thinning compared to NT, while the AS group showed only reduced nasal pRNFL thickness compared to NT. In contrast, García-Medina et al. (2017) reported a significantly thicker total retina (sum of all layers from the RNFL to the photoreceptors (PR)) and total inner retina (sum of all layers from the RNFL to the inner nuclear layer (INL)) at the fovea, as well as a significantly increased inferior pRNFL thickness in 27 children and young adolescents with high-functioning ASD compared to 27 NT. Additionally, they described a trend level elevated inner plexiform layer (IPL) and INL thickness at the fovea and trends of a thicker mean macular thickness (mean thickness of all 9 Early Treatment Diabetic Retinopathy Study (ETDRS) sectors) of the total and inner retina for the autistic adults (García-Medina et al., 2017). They suggested that the increased retinal thickness in ASD might result from parenchyma overgrowth (Bigler et al., 2010) or be related to neuroinflammatory processes such as microglia activation (García-Medina et al., 2017; Nakagawa & Chiba, 2016). Additionally, a positive association between the inferior pRNFL thickness and the composite intelligence quotient (IQ) was detected (García-Medina et al., 2017). A recent study (Garcia-Medina et al., 2020) reported higher optic nerve head (ONH) perfusion density and lower ONH flux at the peripapillary inferior quadrant in 13 autistic adolescents and young adults compared to NT, while the increase in the mean macular and inferior pRNFL thickness in autistic participants remained on trend level.

## Aims of the Study

The objective of the study was to obtain detailed insight into possible structural retinal alterations in autistic adults compared to NT. Based on previous studies, we expected an atypical formation of the pRNFL. Due to inconclusive results of preceding investigations, we wanted to clarify whether the pRNFL is thicker, thinner, or unaltered in autistic adults compared to NT. Moreover, we aimed to compare the structural features of the circumfoveal intraretinal layers (RNFL, ganglion cell layer (GCL), IPL, INL, outer plexiform layer (OPL), outer nuclear layer (ONL), photoreceptor-retinal pigment epithelium (PR + RPE) complex) as well as the overall macular thickness (MT) and volume (MV) between autistic and neurotypical adults. Additionally, we aimed at determining whether there is an association between retinal alterations and the severity of autistic symptoms.

## Methods

### Participants

The study was approved by the local Ethics Committee (Approval ID: 314/18) and conducted in accordance with the Declaration of Helsinki (World Medical Association, 2013).

Thirty-four autistic adults with a diagnosis of Asperger Syndrome according to the 10^th^ revision of the International Classification of Diseases (ICD-10: F84.5) or autism spectrum disorder (ASD) according to the Diagnostic and Statistical Manual of Mental Disorders 5 (DSM-5) criteria were included in the study. The diagnosis was established by experienced specialists in psychiatry and psychotherapy with expertise in the diagnosis of ASD. The diagnostic assessment included the autism spectrum quotient (AQ) (Baron-Cohen et al., 2001), the empathy quotient (EQ) (Baron-Cohen & Wheelwright, 2004) and the Social Responsiveness Scale 2 (SRS-2) (Constantino, 2012), the Beck Depression Inventory (BDI-II) (Beck et al., 1996; Hautzinger et al., 2006) to evaluate comorbid depressive symptoms, the Wender Utah Rating Scale (WURS-k) (Retz-Junginger et al., 2002) for the investigation of comorbid symptoms of attention-deficit/hyperactivity disorder (ADHD) in childhood as well as the Multiple Choice Vocabulary Test (MWT-B) (Lehrl et al., 1995) as measure for crystallized intelligence. The autistic adults overlap with participants who have already been assessed for functional retinal changes using the electroretinogram (ERG) (Friedel et al., 2022b).

Thirty-one NT matched according to sex and age were additionally enrolled in the study. The NT participants had to be without a current or past history of a psychiatric illness. The following questionnaires were collected: the Structured Clinical Interview SCID-I (Wittchen et al., 1997) to rule out axis I psychiatric diseases according to DSM-IV criteria, the SCID-II (Fydrich et al., 1997) to additionally rule out axis II personality disorders, the AQ (Baron-Cohen et al., 2001), EQ (Baron-Cohen & Wheelwright, 2004) and SRS-2 (Constantino, 2012) questionnaire to rule out symptoms of an ASD, the BDI-II (Beck et al., 1996; Hautzinger et al., 2006) questionnaire to exclude a current depressive episode, the WURS-k (Retz-Junginger et al., 2002) to rule out ADHD symptoms in childhood as well as the Symptom Check List-90 (SCL-90) (Derogatis & Savitz, 1999) questionnaire. Moreover, the NT participants completed the MWT-B (Lehrl et al., 1995) questionnaire to assess the IQ.

The following exclusion criteria were defined for all study participants: ophthalmological diseases (with the exception of correctable refraction errors), low visual acuity (< 0.7 decimal visual acuity; measured with the Freiburg Acuity and Contrast Test (Bach, 2007)), myopia lower than − 6 dpt or hyperopia greater than + 6 dpt (Cruz-Herranz et al., 2016), ocular surgery, neurological disorders, diabetes mellitus or arterial hypertension, substance abuse, age < 18 years or > 65 years, bipolar disorder, psychotic symptoms, and insufficient collaboration during OCT imaging.

### Optical Coherence Tomography (OCT)

Data acquisition, processing and reporting were performed in accordance with the “Advised Protocol for OCT Study Terminology and Elements” (APOSTEL) recommendations for OCT studies (Aytulun et al., 2021; Cruz-Herranz et al., 2016).

The SPECTRALIS® OCT (Heidelberg Engineering; mean wavelength of the superluminescent diode: 880 nm) with the Glaucoma Module Premium Edition® (version 6.9.4.0) and the Eye Explorer® software (version 1.10.2.0) were used for retinal imaging of both eyes of the participants. The sample rate for A-scans was 40 kHz in 3.9 µm/pixel depth resolution. Automated papillae and fovea detection were conducted using the implemented anatomic positioning system. The HRA/SPECTRALIS® module (version 6.9.5.0) was used for data export.

**Fig. 1:**
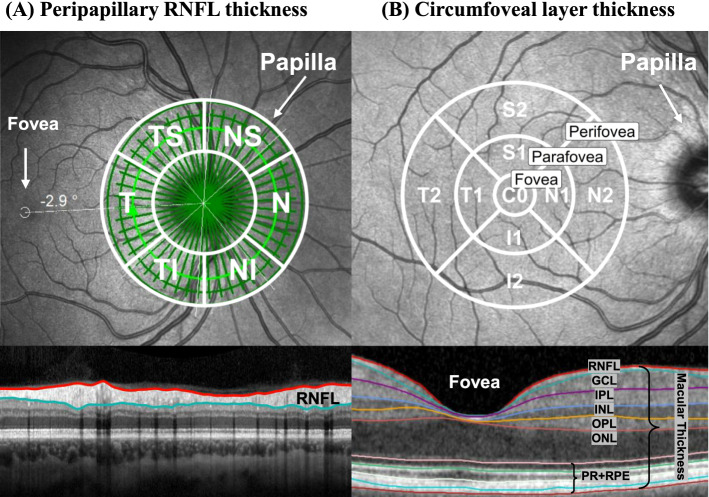
Schematic overview of OCT examinations.** A** Peripapillary RNFL thickness from the 3 circle scans with the 6 sector Garway-Heath grid (white circles and lines) (Garway-Heath et al., 2000).** B** Circumfoveal macular and intraretinal layer thickness measured within the 9 sector ETDRS grid (white circles and lines) (Chew, 1996). Sector data were summarized into total thickness and in 3 foveal regions: fovea, parafovea and perifovea. Abbreviations: GCL = ganglion cell layer; I = inferior; INL = inner nuclear layer; IPL = inner plexiform layer; N = nasal; NI = nasal-inferior; NS = nasal-superior; ONL = outer nuclear layer; OPL = outer plexiform layer; RNFL = retinal nerve fiber layer; PR + RPE = photoreceptor-retinal pigment epithelium complex; S = superior; T = temporal; NI = temporal-inferior; TS = temporal-superior (Friedel et al., 2022a)

The “optic nerve head – radial circle scan” was used to measure the pRNFL thickness. During nasal fixation, 768 A- and 27 B-scans were acquired. Bruch’s membrane opening (BMO) was detected with 24 radial B-scans, 3 B-scans were circle scans with 3.5, 4.1, 4.7 mm diameter around the BMO center (15° scan angle) used for pRNFL evaluation. The global pRNFL thickness as well as the pRNFL thickness data for all tiles of the 6-sector Garway-Heath grid (Garway-Heath et al., 2000) were exported from the device (Fig. 1A).

Circumfoveal thickness and volume data of the macula and the intraretinal layers were obtained using the “posterior pole horizontal scan”. During central fixation, 768 A- and 61 B-scans were taken within a field of 30° × 25° (121 µm distance) around the foveal center (Fig. 1B). The total volume of the macula and all intraretinal layers as well as the thickness data for all tiles of the 9-sector ETDRS grid (Chew, 1996) were exported from the device.

Thickness data from the sectors were summarized into (a) total thickness of the ETDRS grid and (b) 3 foveal regions: fovea (C0; 1 mm), parafovea (S1, N1, I1, T1; 3 mm) and perifovea (S2, N2, I2, T2; 6 mm) (Fig. 1B).

All OCT images were individually checked for artifacts or incorrect layer segmentation and analyzed by an ophthalmologist for pathological findings before data extraction.

### Statistical Analysis

Statistical analysis was performed in “R” (R Core Team, 2021) with RStudio (RStudio Team, 2022) using the “tidyverse”, “broom” and “purrr” packages for data handling (Henry & Wickham, 2020; Robinson et al., 2022; Wickham et al., 2019) and the “ggplot2” package (Wickham, 2016) for graphical representations. When available, data from both eyes were averaged for all participants. If one eye had to be excluded, only the other eye was considered in the analysis.

Since some of the partial datasets showed outliers and slight departures from the normal distribution, robust multiple linear regression models (function “lmrob”; package “robustbase” (Maechler et al., 2021)) were used to estimate differences in OCT measures between NT and autistic adults while controlling for age and sex. The regression formula was defined as:

$$\hat{Y} = \beta_{0} + \beta_{age} *age + \beta_{sex} *sex + \beta_{group} *group$$

The model was based on MM-type estimators (Koller & Stahel, 2011) to robustly predict the effect of group affiliation (β_group_) on the OCT measures ($\widehat{Y}$) while controlling for age and sex as possible confounding variables. Age values were mean centred, and male NT were defined as reference (${\beta }_{0}$). The regression coefficients for group affiliation (β_group_) thus directly indicate the robustly estimated differences of group means after adjusting for age and sex. Cohen’s *d* (Cohen, 1992) as effect size measure for group differences was calculated from the t-statistics of the corresponding group coefficients (“effectsize” package; (Ben-Shachar et al., 2020)). Cohen’s *d* >|0.2| was considered a small effect, *d* >|0.5| a moderate effect, and *d* >|0.8| a large effect.

Spearman correlation coefficients (Best & Roberts, 1975; Hollander & Wolfe, 1973) (*rho*; “rstatix” package (Kassambara, 2021)) were calculated for the autistic adults separately to evaluate associations between alterations in OCT measures and the severity of ASD-related symptoms, as classified by the psychometric instruments: AQ (Baron-Cohen et al., 2001), EQ (Baron-Cohen & Wheelwright, 2004) and SRS-2 (Constantino, 2012).

The significance level was defined as α = 0.05 and adjusted for multiple testing according to the Benjamini-Hochberg (1995) procedure, controlling the false discovery rate (FDR).

The total scores of the WURS-k (Retz-Junginger et al., 2002) and BDI-II (Beck et al., 1996; Hautzinger et al., 2006) were not included as covariates, because of a strong correlation with the predictor variable of group affiliation (no independence). However, to estimate if group differences between ASD and NT persist without possible confounding effects, we subsequently conducted explorative analyses on subsamples of autistic adults. We considered autistic adults (a) without elevated ADHD related symptoms in childhood (WURS-k < 30), (b) without elevated depressive symptoms (BDI-II < 14) and (c) without the intake of psychiatric medication and compared those separately to the NT group.

## Results

### Participants

Table 1 summarizes the demographic and psychometric data for ASD and NT. The mean age in both groups was 35 years (standard deviation: 10 years). Compared to NT, autistic adults showed increased depressiveness according to the BDI-II (Beck et al., 1996; Hautzinger et al., 2006) questionnaire and a higher level of ADHD symptoms in childhood as measured by the WURS-K (Retz-Junginger et al., 2002) questionnaire.

**Table 1:** Demographic and psychometric data

Parameter	Mean (SD) NT (N = 31)	Mean (SD) ASD (N = 34)	*p*-value
Sex (male/female)	19/12	21/13	> .999^#^
Age in years	35 (10)	35 (10)	.963
IQ (MWT-B)	107 (11)	111 (16); Na = 1	.434
SRS-2	32 (13)	104 (27); Na = 2	< .001
AQ	12 (4)	34 (8); Na = 2	< .001
EQ	49 (11)	23 (12)	< .001
BDI-II	3 (3)	18 (13)	< .001
WURS-k	12 (9)	28 (15)	< .001
Psychiatric medication (yes/no)	0/31	20/14	

Group comparisons based on Fishers-Exact- (#) and Wilcoxon-Tests

*ASD* autism spectrum disorder; *AQ* Autism Spectrum Quotient (Baron-Cohen et al., 2001); *BDI-II* Beck Depression Inventory II (Beck et al., 1996; Hautzinger et al., 2006); *EQ* Empathy quotient (Baron-Cohen & Wheelwright, 2004); *N* number; *NT* neurotypical adults; *IQ* intelligence quotient; *MWT-B* Multiple choice vocabulary test (Lehrl et al., 1995); *SRS-2* Social Responsiveness Scale 2 (Constantino, 2012); *SD* standard deviation; *WURS-k* Wender Utah Rating Scale (Retz-Junginger et al., 2002)

Twenty autistic adults took psychiatric medication, while fourteen were unmedicated.

Fifteen took antidepressants (four selective serotonin reuptake inhibitors (SSRI), six serotonin and norepinephrine reuptake inhibitors (SNRI), two bupropion, three tetracyclic and four tricyclic antidepressants, one agomelatine), three methylphenidate, eight low dose atypical neuroleptics for stimulus shielding or as low dose sleeping pills, two mood stabilizers (one lithium, one lamotrigine) and one patient pregabalin.

Four right and five left eyes in the ASD group, as well as three right and three left eyes in the NT group had to be excluded due to low visual acuity (< 0.7 decimal visual acuity) or excessive refraction errors, coincidental pathological findings in the OCT images, inadequate cooperation or insufficient automated segmentation. Thus, 59 eyes from 34 autistic adults and 56 eyes from 31 NT could be included in the final analysis.

### Peripapillary RNFL (pRNFL) Thickness (Optic Nerve Head – Radial Circle Scan)

The robust regression models for the globdal pRNFL thickness from the three circle scans revealed no significant differences in ASD compared to the NT group. However, the coefficients for sex (β_sex_) indicated thicker global pRNFL for female participants in all three models (Table 2). Due to the lack of any group differences, no further post-hoc analyses for the pRNFL thickness data on sector level were performed.

**Table 2:** Arithmetic mean of the global pRNFL thickness and results from the robust regression models

Diameter	Mean (SD)		Results from the robust regression models				
	NT	ASD	Parameter	Coefficients (β)	SE	*p* value	*d*
3.5 mm	102.52 (7.60)	101.43 (8.50)	Age	− 0.07	0.10	.454 (ns)	− 0.19
			Sex (female)	6.65	2.01	.002 (*)	0.86
			Group (ASD)	− 0.51	1.96	.794 (ns)	− 0.07
4.1 mm	87.98 (6.39)	86.7 (7.11)	Age	− 0.06	0.08	.469 (ns)	− 0.19
			Sex (female)	5.25	1.73	.004 (*)	0.78
			Group (ASD)	− 0.94	1.69	.581 (ns)	− 0.14
4.7 mm	76.98 (5.62)	76.17 (5.65)	Age	− 0.05	0.07	.511 (ns)	− 0.17
			Sex (female)	3.56	1.52	.022 (*)	0.61
			Group (ASD)	− 0.46	1.47	.755 (ns)	− 0.08

For both groups and all three scans, data were pooled over the 6 sector tiles of the Garway-Heath grid (Garway-Heath et al., 2000). Results of the robust regression analysis with the parameters coefficients (β), the corresponding standard errors (SE), *p* values, significance level in parentheses (*/ns) and effects sizes (*d*). Neurotypical males were considered as reference (β_0_)

*ASD* autism spectrum disorder; *d* Cohen’s *d; NT* neurotypical adults; *pRNFL* peripapillary retinal nerve fiber layer; *SD* standard deviation; *SE* Standard error of the coefficients; significance level after Benjamini–Hochberg adjustment (Benjamini & Hochberg, 1995) in parentheses: * significant, *ns* not significant

### Circumfoveal Retinal Layer Thicknesses and Volumes (Posterior Pole Horizontal Scan)

#### Total ETDRS Thickness and Volume of the Macula and the Intraretinal Layers

**Fig. 2:**
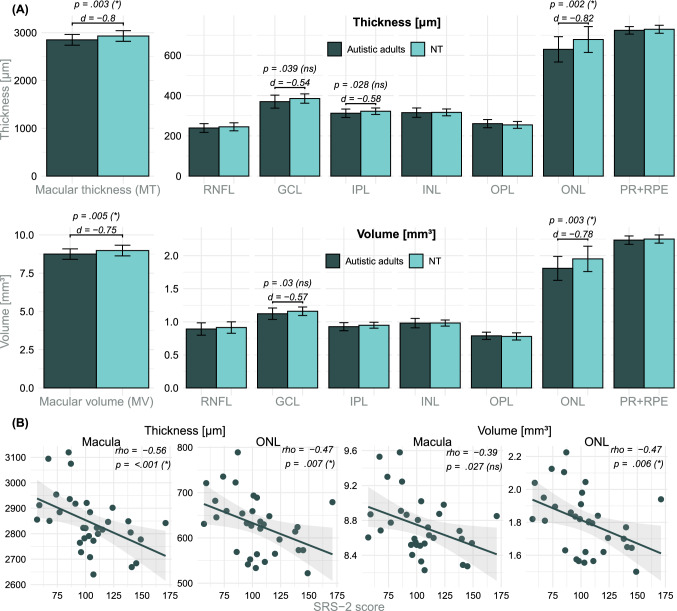
**A** Analysis of the total ETDRS grid data. Average (arithmetic mean and standard deviations as error bars) of the macular and intraretinal layer thicknesses in µm and volumes in mm^3^ for autistic adults and NT. *P* values < .05 of the group coefficients from the robust regression models and their effect sizes (Cohen’s *d*) are depicted. **B** Macular and ONL thickness and volume in relation to the total SRS-2 scores of autistic adults. Spearman’s *rho* and corresponding *p* values with Benjamini-Hochberg (Benjamini & Hochberg, 1995) adjusted significance levels in parentheses (*/ns) are shown. *d* Cohen’s *d*; *GCL* ganglion cell layer; *NT* neurotypical adults; *INL* inner nuclear layer; *IPL* inner plexiform layer; *ns* not significant; *ONL* outer nuclear layer; *OPL* outer plexiform layer; *RNFL* retinal nerve fiber layer; *PR* + *RPE* photoreceptor-retinal pigment epithelium complex; *SRS-2* Social Responsiveness Scale 2; * significant

Figure 2A illustrates the total macular thickness (MT) and volume (MV) as well as the thickness and volume of all intraretinal layers (RNFL, GCL, IPL, INL, OPL, ONL, PR + RPE) for the ASD and NT group.

The regression models revealed a significant reduction in the total MT of autistic adults compared to NT (β_group_ =  − 88 µm (2.99%); *p* = 0.003 (*); *d* =  − 0.8), as well as in the MV (β_group_ =  − 0.26 mm^3^ (2.89%); *p* = 0.005 (*); *d* =  − 0.75). For the intraretinal layers, a significant reduction of the total ONL thickness (β_group_ =  − 53.22 µm (7.75%); *p* = 0.002 (*); *d* =  − 0.82) and ONL volume (β_group_ =  − 0.15 mm^3^ (7.61%); *p* = 0.003 (*); *d* =  − 0.78) were apparent for autistic adults compared to NT.

There was a reduction in the IPL and GCL thickness as well as in the GCL volume of autistic adults, which was not statistically significant after adjustment for multiple testing according to the Benjamini Hochberg procedure (1995). No group differences were found in the other retinal layers (Table 3).

**Table 3:** Mean (SD) thickness [µm] and volume [mm^3^] of the intraretinal layers and the MT and MV

Layer	Arithmetic mean (SD)		Results from the regression models			
	NT	SD	β_group_	SE	*p* value	*d*
Total Volume [μm]						
**MT**	**2930.82 (111.29)**	**2850.29 (112.56)**	**− 88.00**	**28.34**	.003 (*)	**− 0.80**
RNFL	244.97 (19.95)	238.99 (21.66)	− 5.46	5.26	.303 (ns)	− 0.27
GCL	385.42 (23.27)	370.09 (32.27)	− 15.04	7.14	.039 (ns)	− 0.54
IPL	322.36 (16.15)	312.35 (20.88)	− 10.78	4.79	.028 (ns)	− 0.58
INL	316.44 (16.96)	315.34 (23.07)	− 1.40	5.15	.787 (ns)	− 0.07
OPL	254.40 (17.06)	260.57 (20.25)	6.31	5.08	.219 (ns)	0.32
**ONL**	**678.60 (64.26)**	**629.66 (63.02)**	**− 53.22**	**16.70**	.002 (*)	**− 0.82**
PR + RPE	729.44 (20.00)	724.12 (18.74)	− 5.21	4.55	.257 (ns)	− 0.29
Total Volume [mm^3^]						
**MV**	**8.98 (0.35)**	**8.75 (0.34)**	**− 0.26**	**0.09**	.005 (*)	**− 0.75**
RNFL	0.91 (0.09)	0.89 (0.09)	− 0.02	0.02	.318 (ns)	− 0.26
GCL	1.16 (0.06)	1.12 (0.09)	− 0.04	0.02	.030 (ns)	− 0.57
IPL	0.95 (0.04)	0.93 (0.06)	− 0.03	0.01	.047 (ns)	− 0.52
INL	0.98 (0.05)	0.98 (0.07)	0.00	0.01	.812 (ns)	− 0.06
OPL	0.78 (0.05)	0.79 (0.06)	0.01	0.01	.645 (ns)	0.12
**ONL**	**1.95 (0.19)**	**1.81 (0.18)**	**− 0.15**	**0.05**	.003 (*)	**− 0.78**
PR + RPE	2.25 (0.06)	2.24 (0.06)	**− **0.01	0.01	.331 (ns)	− 0.25

Group coefficients (β_group_), standard errors (SE), *p* values (significance level after Benjamini–Hochberg adjustment (Benjamini & Hochberg, 1995) in parentheses) and effects sizes (*d*) from the robust regression models are summarized. Significant differences are highlighted in bold. Male NT were defined as reference (β_0_). Group coefficients (β_group_) represent the robust estimated differences in means [µm/mm^3^] between NT and ASD after adjusting for age and sex

*ASD* autism spectrum disorder; *β*_*group*_ coefficient for the group parameter from the regression model; *d* Cohen’s d; *GCL* ganglion cell layer; *INL* inner nuclear layer; *IPL* inner plexiform layer; *ns* not significant; *MT* macular thickness; *MV* macular volume; *NT* neurotypical adults; *ONL* outer nuclear layer; *OPL* outer plexiform layer; *RNFL* retinal nerve fiber layer; *PR* + *RPE* photoreceptor-retinal pigment epithelium complex; *SD* standard deviation; *SE* standard error of the coefficient; * significant

#### Associations Between Retinal Thinning in Autistic Adults and the Severity of ASD Symptoms

Possible associations between the retinal thinning of the MT, MV and ONL thickness and volume with the severity of ASD symptoms (AQ, EQ, SRS-2) (Baron-Cohen & Wheelwright, 2004; Baron-Cohen et al., 2001; Constantino, 2012) were analyzed calculating Spearman correlation coefficients (*rho*). The significance level was adjusted for the number of tests.

Neither the AQ (Baron-Cohen et al., 2001), nor the EQ (Baron-Cohen & Wheelwright, 2004) scores showed statistically significant correlations with the thickness and volume of the macula and ONL. Significant inverse correlations were found for the total SRS-2 (Constantino, 2012) scores and the MT (*rho* =  − 0.56; *p* < 0.001 (*)), the ONL thickness (*rho* =  − 0.47; *p* = 0.007 (*)) and ONL volume (*rho* =  − 0.47; *p* = 0.006 (*)) (Fig. 2B). The correlation between the SRS-2 score and the MV fell below the threshold for statistical significance after adjustment for multiple testing (*rho* =  − 0.39; *p* = 0.027 (ns)).

#### Subsamples of Autistic Adults Without Comorbid (ADHD Symptoms in Childhood or Depressive) Symptoms or the Intake of Medication

In order to evaluate whether the reduction of the MT, MV and ONL thickness and volume in autistic adults persists independently of comorbid symptoms (ADHD in childhood, depression) or the intake of psychiatric medication, exploratory analyses of subsamples with ASD were conducted (Table 4). The statistically significant reduction in the MT and ONL thickness and volume was also apparent in the ASD subsample without elevated ADHD related symptoms in childhood (N = 19). The MV reduction, however, was no longer significant when compared to NT.

**Table 4:** Exploratory analysis on ASD subsamples without elevated comorbid symptoms or the intake of psychiatric medication

Subsample ASD	Layer & Measure	Results from the regression models comparing ASD subsamples to NT				Correlation with SRS-2 (ASD only)	
		β_group_	SE	*p* value	*d*	*rho*	*p* value
ASD without ADHD symptoms (WURS-k < 30); N = 19	MT [µm]	− 70.65	33.85	**.042**	** − 0.62**	** − 0.59**	**.015**
	ONL thickness [µm]	− 45.34	20.29	**.030**	** − 0.66**	** − 0.64**	**.007**
	MV [mm^3^]	− 0.20	0.11	.080	− 0.53	− 0.39	.126
	ONL volume [mm^3^]	− 0.13	0.06	**.041**	** − 0.62**	** − 0.62**	**.008**
ASD without depressive symptoms (BDI-II < 14); N = 18	MT [µm]	− 95.74	30.53	**.003**	** − 0.94**	** − 0.70**	**.002**
	ONL thickness [µm]	− 41.54	19.76	**.041**	** − 0.63**	** − 0.56**	**.020**
	MV [mm^3^]	− 0.31	0.10	**.003**	** − 0.93**	** − 0.58**	**.015**
	ONL volume [mm^3^]	− 0.12	0.06	.051	− 0.60	** − 0.52**	**.033**
ASD without psychiatric medication; N = 14	MT [µm]	− 118.21	36.03	**.002**	** − 1.02**	** − 0.68**	**.010**
	ONL thickness [µm]	− 58.37	21.38	**.009**	** − 0.85**	** − 0.87**	** < .001**
	MV [mm^3^]	− 0.33	0.12	**.008**	** − 0.87**	** − 0.60**	**.030**
	ONL volume [mm^3^]	− 0.17	0.07	**.012**	** − 0.83**	** − 0.90**	** < .001**

Results from the exploratory regression models for group comparisons: group coefficients (β_group_), standard error (SE), *p *values and effect sizes (*d*), with male neurotypical adults as reference (β_0_). Results from the exploratory correlation analysis for the ASD subsamples: spearman’s *rho* and *p *values for the correlation coefficients. Significant results are highlighted in bold

*ASD* autism spectrum disorder; *BDI-II* Beck Depression Inventory II (Beck et al., 1996; Hautzinger et al., 2006); *β*_*group*_ coefficient for the group parameter from the regression model; *d* Cohen’s *d*; *MT* macular thickness; *MV* macular volume; *NT* neurotypical adults; *ONL* outer nuclear layer; *rho* Spearman’s *rho*; *SE* standard error of the coefficient; *SRS-2* Social Responsiveness Scale 2 (Constantino, 2012); *WURS-k* Wender Utah Rating Scale (Retz-Junginger et al., 2002)

Similarly, the reduction in the MT, MV and the ONL thickness was also observable in the ASD subsample without depressive symptoms (N = 18) when compared to NT, whereas the ONL volume was not significantly reduced.

Comparing the ASD subsample without the intake of psychiatric medication (N = 14) to the NT group, all previously detected reductions in the MT, MV and ONL thickness and volume were still observed.

The subsequent explorative analyses for the associations between the macular and the ONL thicknesses and volumes and the SRS-2 (Constantino, 2012) scores for the ASD subsamples are summarized in Table 4. With the exception of the MV in the ASD subsample without ADHD related symptoms in childhood, the statistically significant inverse associations between the SRS-2 (Constantino, 2012) scores and the macular and ONL thicknesses and volumes were observable in all ASD subsamples.

#### Region and Sector Analyses of the Macular and ONL Thicknesses Within the ETDRS Grid

**Fig. 3:**
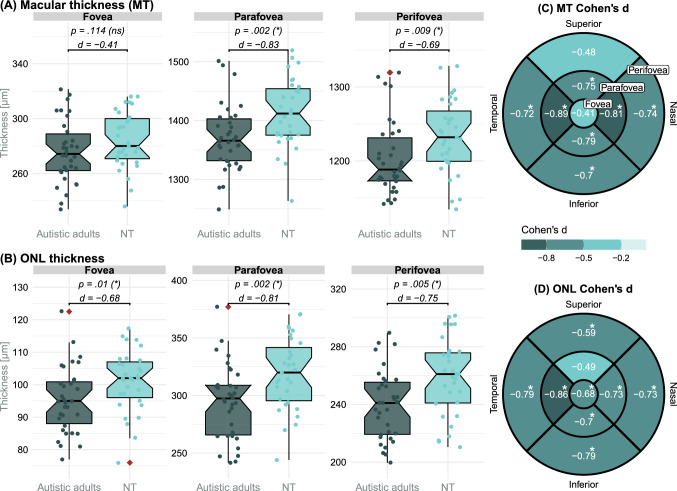
Region and sector analysis of the macular and ONL thickness. The three foveal regions of the MT (**A**) and ONL thickness (**B**) were analyzed in a first step. *P* values for group coefficients (β_group_) from the regression models and corresponding effect sizes (Cohen’s *d*) are depicted. Outliers are indicated by red diamond dots. Benjamini-Hochberg (Benjamini & Hochberg, 1995) adjusted significance level in parentheses (*/ns). Subsequently the MT (**C**) and ONL thickness (**D**) data were analyzed on ETDRS (Chew, 1996) sector level to localize group differences more precisely. Cohen’s *d* for group differences is depicted for the 9 ETDRS (Chew, 1996) sector tiles. The number of tests per analysis (region/sector level) was considered for the correction. *d* Cohen’s *d*; *MT* macular thickness; *ns* not significant; *NT* neurotypical adults; *ONL* outer nuclear layer; * significant

Figure 3A, B illustrates the MT and ONL thickness data for the three foveal regions for both groups. Supplemental Table 1 summarizes the group coefficients of the regression models.

Group coefficients of the robust regression models revealed a significant reduction in the para- (β_group_ =  − 46.14 µm (3.25%); *p* = 0.002 (*); *d* =  − 0.83) and perifoveal (β_group_ =  − 34.22 µm (2.78%); *p* = 0.009 (*); *d* =  − 0.69) MT in ASD.

All three regions of the ONL were statistically significantly reduced in ASD compared to NT (fovea: β_group_ =  − 6.68 µm (6.57%); *p* = 0.01 (*); *d* =  − 0.68; parafovea: β_group_ =  − 26.21 µm (8.11%); *p* = 0.002 (*); *d* =  − 0.81; perifovea: β_group_ =  − 20.15 µm (7.69%); *p* = 0.005 (*); *d* =  − 0.75). The significance level was Benjamini-Hochberg (1995) adjusted according to the number of regression models (6 comparisons).

The subsequent sector analysis revealed, that almost all sectors of the ETDRS grid for the macular and ONL thickness were affected in ASD (Fig. 3C, D and Supplemental Table 1). Only the foveal and the superior-perifoveal sector of the MT as well as the superior-parafoveal sector of the ONL showed no significant thinning in ASD compared to NT. The number of regression models (18 comparisons) was used for Benjamini-Hochberg (1995) adjustment.

The most pronounced reductions in the MT of autistic adults were found within the parafoveal region in the temporal and nasal sectors (large effect; d >|0.8|). The most distinctive thinning in the ONL of autistic adults was observed in the temporal parafovea (large effect; d >|0.8|).

## Discussion

Our study compared thicknesses and volumes of the macula and the different intraretinal layers between autistic adults and NT using OCT. We observed a moderate reduction in the total MT and MV as well as a severe reduction in the total ONL thickness and volume in ASD. The macular thinning was found in the para- and perifoveal region, while alterations in the ONL thickness were detected in all regions (fovea, peri- and parafovea).

In contrast to previous OCT investigations (Emberti Gialloreti et al., 2014; García-Medina et al., 2017), we found no alterations of the RNFL in autistic adults.

While we observed thinning of the MT and ONL in adults with ASD, a previous study by García-Medina et al. (2017) showed greater thickness of the total retina (equivalent to the MT) and the total inner retina (comprising the RNFL, GCL, IPL, INL) at the fovea as well as an elevated inferior pRNFL thickness in ASD. Additionally, the inferior pRNFL thickness was positively associated with the composite IQ of autistic adults (García-Medina et al., 2017).

The different observations may be due to the fact that the preceding study examined autistic children and adolescents (García-Medina et al., 2017), while we focused on adults. The retinal thickening in children (García-Medina et al., 2017), trends towards an elevated MT and pRNFL thickness in adolescents and young adults with ASD (Garcia-Medina et al., 2020) and the finding of retinal thinning in adults with ASD in our and a preceding study (Emberti Gialloreti et al., 2014) might complement observations from neuroimaging studies. While there is consensus on early brain overgrowth during infancy (Ecker et al., 2015; Hazlett et al., 2011; Prigge et al., 2021) followed by an arrested growth and normalization in brain volume from adolescence to late middle age (Courchesne et al., 2011; Ecker et al., 2015; Prigge et al., 2021), alterations in cerebral structures in adulthood in ASD are under debate. Although some studies pointed towards a reduced brain volume in autistic adults (Courchesne et al., 2011; Ecker et al., 2015), others reported normal brain (Denier et al., 2022; Riedel et al., 2014) and gray matter (Prigge et al., 2021) volumes as well as an unaltered cortical thickness in autistic adults with ASD (Maier et al., 2018). Likewise, we found no reductions in the pRNFL, RNFL, GCL, IPL, INL, OPL and PR + RPE, which were reported to be positively associated with the total brain, the total gray and white matter volumes (Mauschitz et al., 2022). The thickness of the ONL –comprising the cell bodies of the photoreceptors, representing the first step in the retinal afferent path– seemed not to be correlated with any of these cerebral structures (Mauschitz et al., 2022). The reasons for the retinal alterations observed in adults with ASD, however, have not yet been clarified or directly compared to brain imaging data. As potential underlying correlates, neuroinflammatory processes (Nakagawa & Chiba, 2016) or a modification in the programmed cell death (Wei et al., 2014) within the retina have been discussed.

It has to be stressed that assumptions with respect to the precise link between retinal and global neuronal network abnormalities in ASD remain speculative. One could speculate that the volume loss of the retinal ONL is due to migratory or other neurodevelopmental network perturbations that reflect synchronously occurring neurodevelopmental perturbations in isocortical networks.

Alternatively, the ONL abnormalities could reflect a shift in GABAergic and glutamatergic signaling in ASD, as postulated by the excitatory/inhibitory imbalance hypothesis (E/I imbalance hypothesis), given that both neurotransmitters also play a central role at a retinal level (Rubenstein & Merzenich, 2003; Tebartz van Elst et al., 2014). In this framework, the retina was examined in a prenatally exposed valproic acid autism mouse model in a previous study (Guimarães-Souza et al., 2019), because sensoric hypo-/hypersensitivity to visual stimuli is a well known phenomenon in ASD. The retina, as part of the CNS, mainly uses GABA and glutamate to modulate and transmit visual signals (Wu, 2010). Guimarães-Souza et al. (2019) detected reduced GABA expression and increased mGluR5 in the retinas of early adolescent mice prenatally exposed to valproic acid (Guimarães-Souza et al., 2019), a finding that relates well to the proposed E/I imbalance hypothesis of ASD (Rubenstein & Merzenich, 2003).

While we detected structural retinal alterations of the ONL and MT, we found no functional retinal changes in an ERG study in an overlapping sample of autistic adults (Friedel et al., 2022b). However, how and whether functional and structural retinal changes are associated and related to the E/I imbalance hypothesis needs to be further investigated in combined ophthalmologic and neuroimaging studies in the future.

We detected a significant inverse correlation between the severity of ASD-related symptoms and presence of social impairment, measured by the total SRS-2 score (Constantino, 2012), and the thickness and volume of the ONL. Based on these association, future longitudinal studies should investigate whether alterations in retinal layer thickness could represent a suitable biomarker for ASD. It remains to be clarified how the thickness of the ONL develops during the course of the disorder, and whether it might represent a state or a possible trait marker.

### Limitations

The cross-sectional design of our study does not allow conclusions on whether the detected alterations in the ONL represent a state or a trait marker. The results require replication in a larger sample, however, the current study examined a comparable number of autistic adults using OCT as previous studies. Based on the psychometric assessments, autistic adults had significantly elevated symptoms of depression and ADHD in childhood when compared to NT, a potential source of bias. However, the reduction in the MT and ONL thickness and volume was also significant in the subsample of autistic adults without elevated scores of ADHD related symptoms in childhood. Likewise, the MT, MV and ONL thickness reduction was also detected in the ASD subgroup without depressive symptoms.

Although possible confounding effects of psychiatric medication cannot be ruled out, the analysis of the ASD subsample without psychiatric medication revealed, that all previously observed reductions in the MV, MT and ONL thickness and volume were also apparent in unmedicated autistic adults. Therefore, it is unlikely that the retinal thinning is due to a medication effect.

## Conclusions

Retinal layer thicknesses and volumes in thirty-four autistic adults and thirty-one NT adults were investigated and compared with OCT. We detected a reduction of the total MT, MV as well as of the total ONL thickness and volume. Macular thinning was restricted to the para- and perifoveal region, whereas ONL thinning was observed in all regions (fovea, peri- and parafovea) in autistic adults.

Further studies are required to clarify whether these structural retinal alterations are already evident in early childhood or manifest during the course of the disease. To gain a more comprehensive picture of ASD, future investigations may evaluate how retinal and cerebral alterations are interconnected, whether the structural deficits of the retina are associated with atypical retinal functioning and how other parameters such as medication intake may play a role. It would be desirable to analyze a larger sample of autistic adults across different age spans and additionally include a larger cohort of medication naïve participants.

### Supplementary Information

Below is the link to the electronic supplementary material.

Supplementary file1 (DOCX 21 kb)

## Data Availability

Acquired OCT and demographic data as well as R code for data transformation and statistical analysis, used for this study, will be available from the corresponding author and EF on request.
