# Supplementary material for: Retinal Thinning in Adults with Autism Spectrum Disorder
Source: J Autism Dev Disord. 2022 Dec 23;54(3):1143–56. doi: 10.1007/s10803-022-05882-8 (PMC10907434; doi:10.1007/s10803-022-05882-8)
Supplement: Supplementary file 1 — Supplementary file1 (DOCX 21 kb) [file 10803_2022_5882_MOESM1_ESM.docx]

**Supplemental information**

| **Supplemental Table 1: Comparisons of the MT and ONL thickness between neurotypical adults (NT) and autistic adults (ASD) on region and sector level.** | | | | | | | |
| --- | --- | --- | --- | --- | --- | --- | --- |
|  |  | **Arithmetic mean (SD)** | | **Results from the regression models** | | | |
| **Layer** | **Region** | **NT (n=31)** | **ASD (n=34)** | **𝛽_group_** | **SE** | ***p* value** | ***d*** |
| MT | Fovea | 284.08 (20.70) | 276.18 (22.62) | −8.84 | 5.51 | .114 (ns) | −0.41 |
|  | Parafovea | 1413.45 (58.58) | 1370.79 (56.11) | −46.14 | 14.24 | **.002 (*)** | −0.83 |
|  | Perifovea | 1233.29 (50.11) | 1203.32 (49.51) | −34.22 | 12.67 | **.009 (*)** | −0.69 |
| ONL | Fovea | 101.08 (9.47) | 95.31 (10.08) | −6.68 | 2.53 | **.010 (*)** | −0.68 |
|  | Parafovea | 318.81 (31.37) | 294.57 (32.14) | −26.21 | 8.27 | **.002 (*)** | −0.81 |
|  | Perifovea | 258.71 (26.7) | 239.78 (24.01) | −20.15 | 6.88 | **.005 (*)** | −0.75 |
| **Layer** | **Sector** | **NT (N=31)** | **ASD (N=34)** | **𝛽_group_** | **SE** | ***p* value** | ***d*** |
| MT | C0 | 284.08 (20.70) | 276.18 (22.62) | −8.84 | 5.51 | .114 (ns) | −0.41 |
|  | I1 | 354.52 (14.88) | 344.09 (13.33) | −11.15 | 3.61 | **.003 (*)** | −0.79 |
|  | I2 | 299.81 (11.14) | 292.41 (11.96) | −8.28 | 3.02 | **.008 (*)** | −0.70 |
|  | N1 | 357.64 (15.59) | 346.38 (15.94) | −12.13 | 3.85 | **.003 (*)** | −0.81 |
|  | N2 | 330.13 (15.95) | 319.88 (14.73) | −11.40 | 3.96 | **.005 (*)** | −0.74 |
|  | S1 | 357.23 (15.32) | 347.03 (14.19) | −10.84 | 3.73 | **.005 (*)** | −0.75 |
|  | S2 | 309.14 (13.10) | 304.07 (13.43) | −6.21 | 3.29 | .064 (ns) | −0.48 |
|  | T1 | 344.06 (14.43) | 333.29 (13.93) | −11.98 | 3.44 | **<.001 (*)** | −0.89 |
|  | T2 | 294.21 (11.91) | 286.96 (13.19) | −8.74 | 3.11 | **.007 (*)** | −0.72 |
| ONL | C0 | 101.08 (9.47) | 95.31 (10.08) | −6.68 | 2.53 | **.010 (*)** | −0.68 |
|  | I1 | 76.36 (7.02) | 70.24 (9.26) | −5.94 | 2.16 | **.008 (*)** | −0.70 |
|  | I2 | 59.47 (5.82) | 54.91 (5.55) | −4.82 | 1.55 | **.003 (*)** | −0.79 |
|  | N1 | 84.61 (8.62) | 78.81 (8.74) | −6.45 | 2.25 | **.006 (*)** | −0.73 |
|  | N2 | 66.27 (7.53) | 61.31 (6.81) | −5.47 | 1.93 | **.006 (*)** | −0.73 |
|  | S1 | 78.39 (9.23) | 72.85 (11.60) | −5.29 | 2.74 | .058 (ns) | −0.49 |
|  | S2 | 68.45 (7.46) | 64.28 (7.05) | −4.47 | 1.94 | **.025 (*)** | −0.59 |
|  | T1 | 79.45 (8.15) | 72.68 (8.31) | −7.18 | 2.14 | **.001 (*)** | −0.86 |
|  | T2 | 64.52 (6.70) | 59.28 (6.27) | −5.35 | 1.75 | **.003 (*)** | −0.79 |
| The arithmetic means (SD) and the results of the robust regression models are depicted for the three foveal regions and the single sectors of the ETDRS grid (Chew 1996). Group coefficients (𝛽_group_) from the robust regression models (𝛽_0_= male NT), standard errors (SE), *p* values with adjusted significance level in parentheses (*/ns) and effects sizes (*d*) are depicted. Benjamini-Hochberg procedure (Benjamini and Hochberg 1995) was applied for the region and sector analyses separately, in order to control the false discovery rate. The number of tests per analysis (region or sector level) was considered for the correction. Significant results are highlighted in bold.  Abbreviations: ASD = autism spectrum disorder; 𝛽_group_ = coefficient for the group parameter from the regression model; C0 = fovea; *d* = Cohen’s *d*; I = inferior, MT = macular thickness; N = nasal; n = Number NT = neurotypical adults; ns = not significant; ONL = outer nuclear layer; S = superior; SE = standard error of the coefficient; T = temporal; * = significant. | | | | | | | |

Benjamini Y, Hochberg Y (1995) Controlling the False Discovery Rate: A practical and powerful approach to multiple testing. Journal of the Royal Statistical Society Series B (Methodological) 57:289–300

Chew EY (1996) Association of elevated serum lipid levels with retinal hard exudate in diabetic retinopathy: Early Treatment Diabetic Retinopathy Study (ETDRS) Report 22. Arch Ophthalmol 114:1079. https://doi.org/10.1001/archopht.1996.01100140281004
